# Muscle Homeostasis and Regeneration: From Molecular Mechanisms to Therapeutic Opportunities

**DOI:** 10.3390/cells9092033

**Published:** 2020-09-04

**Authors:** Antonio Musarò

**Affiliations:** Laboratory affiliated to Istituto Pasteur Italia—Fondazione Cenci Bolognetti, DAHFMO-Unit of Histology and Medical Embryology, Sapienza University of Rome, Via Antonio Scarpa, 14, 00161 Rome, Italy; antonio.musaro@uniroma1.it; Tel.: +39-0649766956; Fax: +39-06-4462854

**Keywords:** muscle homeostasis, muscle regeneration, satellite cells, stem cells, FAPs, tissue niche, growth factors, inflammatory response, muscle pathology, aging

## Abstract

The capacity of adult muscle to regenerate in response to injury stimuli represents an important homeostatic process. Regeneration is a highly coordinated program that partially recapitulates the embryonic developmental program and involves the activation of the muscle compartment of stem cells, namely satellite cells, as well as other precursor cells, whose activity is strictly dependent on environmental signals. However, muscle regeneration is severely compromised in several pathological conditions due to either the progressive loss of stem cell populations or to missing signals that limit the damaged tissues from efficiently activating a regenerative program. It is, therefore, plausible that the loss of control over these cells’ fate might lead to pathological cell differentiation, limiting the ability of a pathological muscle to sustain an efficient regenerative process. This Special Issue aims to bring together a collection of original research and review articles addressing the intriguing field of the cellular and molecular players involved in muscle homeostasis and regeneration and to suggest potential therapeutic approaches for degenerating muscle disease.

The regeneration of adult tissues is a highly coordinated process that partially recapitulates the embryonic developmental program. The myogenic program involves an interplay between the intrinsic program of muscle lineage specification and extrinsic influence, such as innervation and growth factor activity. Along with a set of four key transcription factors, namely MyoD, Myf5, Myogenin, MRF4, other factors have been characterized that play a critical role during the different stages of muscle development and regeneration. The transcription factor NF-Y is an evolutionarily conserved heterotrimer formed by the sequence-specific NF-YA and the Histone Fold Domain—HFD—NF-YB/N. Libetti et al., analyzed the role of the two NF-YA isoforms in myogenic program, proposing that NF-YAl, but not NF-YAs, maintains muscle commitment by indirectly regulating Myogenin and MyoD expression in C2C12 cells [1]. The myogenic program is also typically regulated by multiple signaling pathways and by the interaction of several extracellular matrix components with muscle stem cells. Lee et al., demonstrated that Thyroid hormones (THs, thyroxin; T4 and triiodothyronine; T3) regulate the expression of various proteins crucial for muscle development and contractility [2], whereas Torrente et al., discussed the role of insulin-like growth factor axis on fetal and post-natal growth [3]. The characterization of molecular mechanisms involved in the myogenic program also provides a roadmap for recapitulating skeletal myogenesis in vitro from pluripotent stem cells (PSCs). Baci et al., established a reliable protocol to induce the myogenic differentiation of induced pluripotent stem cells (iPSCs), generated from pericytes and fibroblasts, exploiting skeletal muscle-derived extracellular vesicles (EVs), in combination with chemically defined factors [4]. This genetic integration-free approach generated functional skeletal myotubes, maintaining the engraftment ability in vivo. The authors demonstrated that EVs can act as biological “shuttles” to deliver specific bioactive molecules for a successful transgene-free differentiation offering new opportunities for disease modeling and regenerative approaches [4].

The life-long maintenance of muscle tissue has been the objective of numerous studies employing a variety of approaches. It is generally accepted that cumulative failure to repair damage related to an overall decrease in anabolic processes is a primary cause of functional impairment in muscle. Muscle regeneration occurs in five interrelated and time-dependent phases, namely degeneration (necrosis), inflammation, regeneration, remodeling, and maturation/functional repair [5]. Although the phases of muscle regeneration are similar in different organisms (e.g., mouse, rat, human) and after different types of damage/trauma, the kinetics and amplitude of each phase are different in each organism and may depend on the extent of damage and the damage model used. Animals adopt different basic strategies of regeneration that include the activation of adult stem cells, the dedifferentiation of preexisting cells, and/or the proliferation of differentiated cells. This diversity of mechanisms is still widely understudied and underexploited for biomedical applications.

Forcina et al., provided an exhaustive overview about the general aspects of muscle regeneration and discussed the different approaches to study the interrelated and time-dependent phases of muscle healing [5], whereas Zullo et al., discussed, in an interesting review, the insights into the evolutionary aspect of muscle regeneration [6]. In these reviews, the authors integrated the principles of the physiologic muscle regeneration with a technical approach, reporting key experimental methods and markers employed to study cellular and molecular interactors dominating each stage of muscle healing [5] and provided an outline of main animals’ clades, muscle types, their development, homeostasis, and regeneration abilities highlighting what is known of their molecular mechanisms [6]. Moreover, the review by Poovathumkadavil and Jagla [7] discussed how the Drosophila model could help understand the mechanisms of muscle homeostasis and regeneration, discussing the genetic control mechanisms of muscle contraction, development, and homeostasis with particular emphasis on the contractile unit of the muscle, the sarcomere.

The maintenance of an efficient regeneration process is guaranteed by both satellite cells’ niche environment and satellite cells’ pool. By disrupting either one of the two or both, the impairment in muscle regeneration suddenly happens, as likely occurs in many muscular dystrophies. Indeed, one of the critical points that remain to be addressed is why skeletal muscle fails to regenerate under pathological conditions. Either the resident muscle stem cells drastically decrease during aging and in several degenerative diseases or perhaps the pathological muscle is a prohibitive environment for stem cells activation and function. Although we lack definitive answers, several studies suggested that with age or under pathological conditions, the systemic environment impinges the activity of satellite cells and stimulates fibrotic accumulation [8].

Muscle homeostasis and regeneration are indeed severely impinged in several pathologic conditions such as sarcopenia, cachexia, and muscle dystrophies.

In the review by Pratt et al. [9] the authors aimed to identify genetic variants known to be associated with muscle phenotypes relevant to sarcopenia, the progressive deterioration in skeletal muscle mass, strength, and physical function with advancing age. The authors, interrogating PubMed, Embase and Web, using pre-defined search terms such as “aging”, “sarcopenia”, “skeletal muscle”, “muscle strength” and “genetic association”, identified the genetic variants associated with muscle phenotypes relevant to sarcopenia in humans; thus, the review might help to further illuminate the genetic basis of sarcopenia. Heterotopic ossification (HO) is another dysregulation process of skeletal muscle homeostasis and regeneration, which results in mature bone formation in atypical locations. Pulik et al., made the survey of cells responsible for heterotopic ossification development in skeletal muscles [10].

Satellite cells (SCs) are the main actors of myofiber regeneration after damage. Nevertheless, successful muscle healing requires the participation of additional cell types that directly or indirectly contribute to this process. SC activity is known to be influenced by signals deriving from the surrounding environment and by interactions with other cellular components of muscle niche. Petrilli et al., provided a comprehensive picture of the dynamics of the major cell populations that sensed and responded to acute damage in wild type mice and in a mouse model of Duchenne muscular dystrophy, a genetic disease characterized by impaired regenerative process and muscle wasting [11].

One of the mechanisms by which cells communicate with each other involves a conserved delivery system based on the generation and release of extracellular vesicles (EVs). These vesicles transfer information between cells through several categories of cargo-enriched biomolecules (i.e., proteins, lipids, nucleic acids, and sugars), each of them selectively influencing different cellular domains. Picca et al., demonstrated that older adults with physical frailty and sarcopenia show increased levels of circulating small extracellular vesicles with a specific mitochondrial signature [12].

Muscle wasting disorders, including sarcopenia and muscular dystrophies, are also characterized by the gradual replacement of muscle fibers by adipose and fibrotic tissue, which represent critical components of a hostile microenvironment/niche. Transforming Growth Factor β (TGF-β) is known for its role in the regulation of skeletal muscle size as well as fibrosis. Hillege et al. [13] assessed the time-dependent effects of TGF-β signaling and downstream signaling on the expression of myogenic, atrophic, and fibrotic genes in both myoblasts and myotubes. The authors showed that TGF-β inhibits myogenic gene expression in both myoblasts and myotubes but does not affect myotube size in vitro. Most importantly, TGF-β regulates the expression of fibrotic genes in both myoblasts and myotubes in a time-dependent manner [13].

Recent studies on the factors involved in skeletal muscle growth, differentiation, homeostasis, and regeneration have provided new insights into the function of these signaling molecules in muscle and suggest promising new avenues for systemic as well as local intervention in the defects associated with many muscle pathologies. Thus, skeletal muscle repair/regeneration may benefit by the treatments with factors that favor regenerative myogenesis and support the robustness of regenerated myofibers.

The inflammatory response of injured skeletal muscle plays an important and critical role in muscle homeostasis and regeneration and involves the recruitment of specific myeloid cell populations within the injured area. Thus, the inflammatory response is a coordinate process that must be finely regulated to obtain an efficient regenerative process, and the perturbed spatial distribution of inflammatory cells, altered identity of the inflammatory infiltrate (cell type and magnitude of influx) and disrupted temporal sequence results in a persistent rather than resolved inflammatory phase. The transcription factor Nfix, a member of the nuclear factor I (Nfi) family, plays a pivotal role during muscle development, regeneration and in the progression of muscular dystrophies. Saclier, et al., showed that *Nfix* is mainly expressed by anti-inflammatory macrophages [14]. Upon acute injury, mice deleted for *Nfix* in myeloid line displayed a significant defect in the process of muscle regeneration. Indeed, Nfix protein is involved in the macrophage phenotypical switch and macrophages lacking *Nfix* failed to adopt an anti-inflammatory phenotype and interact with myogenic cells.

Cappellari et al., taking Duchenne muscular dystrophy as a paradigm of defect in muscle regeneration, reviewed the main effects of drugs on regeneration biomarkers to assess whether targeting pathogenic events can help to protect niche homeostasis and enhance regeneration efficiency other than protecting newly formed fibers from further damage [15]. Squecco et al., reported the beneficial role of Platelet-Rich Plasma (PRP) treatment owing to PRP pro-myogenic and anti-fibrotic effects [16]. The main relevance of this study was the contribution toward defining the molecular and functional mechanisms regulating TGF-β1-induced fibroblast–myofibroblast transition, highlighting the role of Gap Junctions in this process as well as the involvement of voltage-dependent connexin isoform, namely Cx43. Leduc-Gaudet et al., analyzed the effect of Parkin on disease model, demonstrating that Parkin overexpression prevents sepsis-induced skeletal muscle atrophy, likely by improving mitochondrial quality and contents [17].

The study by Soendenbroe et al., supported the role of exercise in maintaining NMJ stability, even in elderly inactive individuals [18], whereas Jin and co-workers demonstrated that the functions of Lysine (Lys), the first limiting essential amino acid for mammals, in muscle mass accumulation are mediated by satellite cells and the mTORC1 pathway [19].

The maintenance of skeletal muscle mass plays a critical role in health and quality of life. Jorgenson et al., discussed how mechanical loads are one of the most potent regulators of muscle mass [20]. The authors have summarized the major structural adaptations that have been implicated in the mechanical load-induced growth of skeletal muscle and have also considered whether each of these adaptations makes a substantive contribution to the overall growth process [20].

Homeostasis represents one of the most important and critical parameters of adult tissues, including skeletal muscle, and it is defined as the capability of a system to maintain a constant state of complexity and order in a dynamic equilibrium. Interestingly, skeletal muscle is well-maintained in hibernators during hibernation, a unique survival strategy exhibited by various mammals in order to cope with adverse environments in winter, during which hibernators not only face the challenge of prolonged skeletal muscle inactivity, but also deal with other stresses, including hypoxia, fasting, and repeated ischemia-reperfusion during the torpor-arousal cycle.

The maintenance of cytoplasmic calcium (Ca^2+^) homeostasis is important for the preservation of a normal structure and function of skeletal muscle fibers. Skeletal muscle inactivity can trigger Ca^2+^ homeostasis disturbance, often characterized by cytoplasmic Ca^2+^ overload, leading to protein degradation and cell apoptosis, both involved in skeletal muscle loss. Zhang et al., demonstrated that under extreme conditions, such as low temperature, low metabolism, and prolonged hindlimb inactivity during hibernation, hibernating ground squirrels still possess the ability to maintain intracellular Ca^2+^ homeostasis [21]. Therefore, maintaining intracellular Ca^2+^ homeostasis and avoiding skeletal muscle injury caused by its disturbance appear to be priority strategies employed by hibernating squirrels to cope with the various stresses induced during the torpor–arousal cycle. In addition, Malatesta et al., demonstrated that during hibernation, satellite cell nuclei maintain similar transcription and splicing activity as in euthermia, indicating an unmodified status during immobilization and hypometabolism [22]. Skeletal muscle preservation during hibernation is presumably not due to satellite cells activation, but rather to the maintenance of some functional activity in myofibers that is able to counteract muscle wasting

We believe that the papers in this Special Issue, each addressing a specific aspect of muscle homeostasis and regeneration under physiopathologic conditions, will help us to better understand the underlying mechanisms and will help to design more appropriate therapeutic approaches to improve muscle regeneration and to counteract muscle diseases.

## References

[1] Libetti D., Bernardini A., Sertic S., Messina G., Dolfini D., Mantovani R. (2020). The Switch from NF-YAl to NF-YAs Isoform Impairs Myotubes Formation. Cells.

[2] Lee E.J., Shaikh S., Choi D., Ahmad K., Baig M.H., Lim J.H., Lee Y., Park S.J., Kim Y.-W., Park S.-Y. (2019). Transthyretin Maintains Muscle Homeostasis through the Novel Shuttle Pathway of Thyroid Hormones during Myoblast Differentiation. Cells.

[3] Torrente Y., Bella P., Tripodi L., Villa C., Farini A. (2020). Role of Insulin-Like Growth Factor Receptor 2 across Muscle Homeostasis: Implications for Treating Muscular Dystrophy. Cells.

[4] Baci D., Chirivì M., Pace V., Maiullari F., Milan M., Rampin A., Somma P., Presutti D., Garavelli S., Bruno A. (2020). Extracellular Vesicles from Skeletal Muscle Cells Efficiently Promote Myogenesis in Induced Pluripotent Stem Cells. Cells.

[5] Forcina L., Cosentino M., Musarò A. (2020). Mechanisms Regulating Muscle Regeneration: Insights into the Interrelated and Time-Dependent Phases of Tissue Healing. Cells.

[6] Zullo L., Bozzo M., Daya A., Di Clemente A., Mancini F.P., Megighian A., Nesher N., Röttinger E., Shomrat T., Tiozzo S. (2020). The Diversity of Muscles and Their Regenerative Potential across Animals. Cells.

[7] Poovathumkadavil P., Jagla K. (2020). Genetic Control of Muscle Diversification and Homeostasis: Insights from Drosophila. Cells.

[8] Scicchitano B.M., Sica G., Musarò A. (2016). Stem Cells and Tissue Niche: Two Faces of the Same Coin of Muscle Regeneration. Eur. J. Transl. Myol..

[9] Pratt J., Boreham C., Ennis S., Ryan A.W., De Vito G. (2019). Genetic Associations with Aging Muscle: A Systematic Review. Cells.

[10] Pulik Ł., Mierzejewski B., Ciemerych M.A., Brzóska E., Łęgosz P. (2020). The Survey of Cells Responsible for Heterotopic Ossification Development in Skeletal Muscles-Human and Mouse Models. Cells.

[11] Petrilli L.L., Spada F., Palma A., Reggio A., Rosina M., Gargioli C., Castagnoli L., Fuoco C., Cesareni G. (2020). High-Dimensional Single-Cell Quantitative Profiling of Skeletal Muscle Cell Population Dynamics during Regeneration. Cells.

[12] Picca A., Beli R., Calvani R., Coelho-Júnior H.J., Landi F., Bernabei R., Bucci C., Guerra F., Marzetti E. (2020). Older Adults with Physical Frailty and Sarcopenia Show Increased Levels of Circulating Small Extracellular Vesicles with a Specific Mitochondrial Signature. Cells.

[13] Hillege M.M.G., Galli Caro R.A., Offringa C., de Wit G.M.J., Jaspers R.T., Hoogaars W.M.H. (2020). TGF-β Regulates Collagen Type I Expression in Myoblasts and Myotubes via Transient Ctgf and Fgf-2 Expression. Cells.

[14] Saclier M., Lapi M., Bonfanti C., Rossi G., Antonini S., Messina G. (2020). The Transcription Factor Nfix Requires RhoA-ROCK1 Dependent Phagocytosis to Mediate Macrophage Skewing during Skeletal Muscle Regeneration. Cells.

[15] Cappellari O., Mantuano P., De Luca A. (2020). “The Social Network” and Muscular Dystrophies: The Lesson Learnt about the Niche Environment as a Target for Therapeutic Strategies. Cells.

[16] Squecco R., Chellini F., Idrizaj E., Tani A., Garella R., Pancani S., Pavan P., Bambi F., Zecchi-Orlandini S., Sassoli C. (2020). Platelet-Rich Plasma Modulates Gap Junction Functionality and Connexin 43 and 26 Expression During TGF-β1-Induced Fibroblast to Myofibroblast Transition: Clues for Counteracting Fibrosis. Cells.

[17] Leduc-Gaudet J.P., Mayaki D., Reynaud O., Broering F.E., Chaffer T.J., Hussain S.N.A., Gouspillou G. (2020). Parkin Overexpression Attenuates Sepsis-Induced Muscle Wasting. Cells.

[18] Soendenbroe C., Bechshøft C.J.L., Heisterberg M.F., Jensen S.M., Bomme E., Schjerling P., Karlsen A., Kjaer M., Andersen J.L., Mackey A.L. (2020). Key Components of Human Myofibre Denervation and Neuromuscular Junction Stability are Modulated by Age and Exercise. Cells.

[19] Jin C.L., Ye J.L., Yang J., Gao C.Q., Yan H.C., Li H.C., Wang X.Q. (2019). mTORC1 Mediates Lysine-Induced Satellite Cell Activation to Promote Skeletal Muscle Growth. Cells.

[20] Jorgenson K.W., Phillips S.M., Hornberger T.A. (2020). Identifying the Structural Adaptations that Drive the Mechanical Load-Induced Growth of Skeletal Muscle: A Scoping Review. Cells.

[21] Zhang J., Li X., Ismail F., Xu S., Wang Z., Peng X., Yang C., Chang H., Wang H., Gao Y. (2019). Priority Strategy of Intracellular Ca2+ Homeostasis in Skeletal Muscle Fibers During the Multiple Stresses of Hibernation. Cells.

[22] Malatesta M., Costanzo M., Cisterna B., Zancanaro C. (2020). Satellite Cells in Skeletal Muscle of the Hibernating Dormouse, a Natural Model of Quiescence and Re-Activation: Focus on the Cell Nucleus. Cells.

